# Supplementary material for: Stealth Luminescent Organic Nanoparticles Made from Quadrupolar Dyes for Two-Photon Bioimaging: Effect of End-Groups and Core
Source: Molecules. 2022 Mar 29;27(7):2230. doi: 10.3390/molecules27072230 (PMC9000497; doi:10.3390/molecules27072230)
Supplement: Supplementary file 1 [file molecules-27-02230-s001.zip › molecules-1624376-supplementary.pdf]

Stealth Luminescent Organic Nanoparticles Made from Quadrupolar Dyes for Two-Photon Bioimaging: Effect of End-Groups and Core.

Morgane Rosendale †, Jonathan Daniel †, Frédéric Castet, Paolo Pagano, Jean-Baptiste Verlhac and Mireille Blanchard-Desce *

Université Bordeaux, CNRS, Bordeaux INP, ISM, UMR 5255, F-33400 Talence, France;
* Correspondence: mireille.blanchard-desce@u-bordeaux.fr
† These authors contributed equally

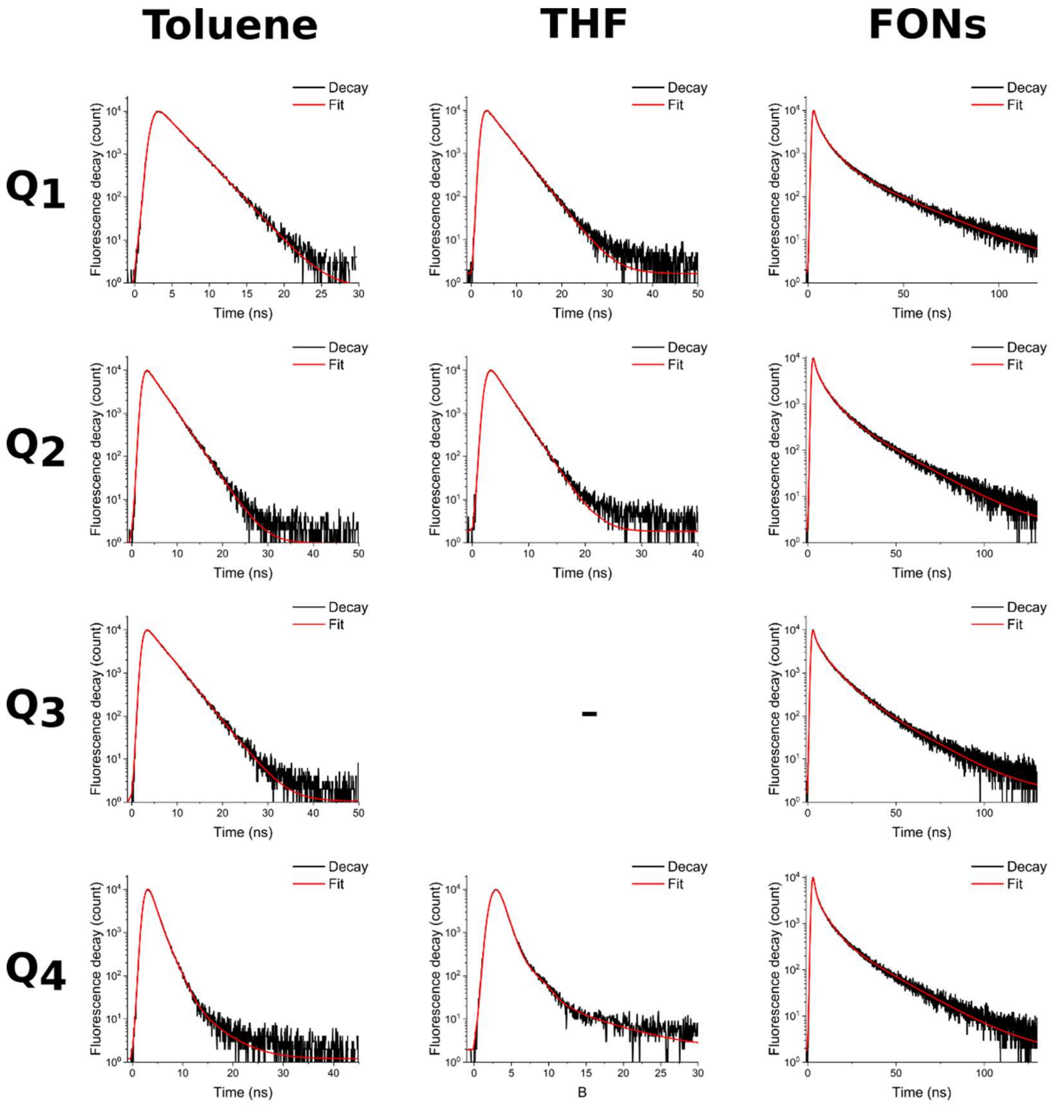

Figure S1. Example fluorescence decays (black) and their fits (red) of dyes **Q1-Q4** in toluene (left), THF (middle) and as FONs in water (right).
